# Genomic and Transcriptomic Analysis Reveals Cuticular Protein Genes Responding to Different Insecticides in Fall Armyworm *Spodoptera frugiperda*

**DOI:** 10.3390/insects12110997

**Published:** 2021-11-05

**Authors:** Jia-Ying Zhu, Lu Li, Kai-Ran Xiao, Shu-Qi He, Fu-Rong Gui

**Affiliations:** 1 Key Laboratory of Forest Disaster Warning and Control of Yunnan Province, Southwest Forestry University, Kunming 650224, China; lilu0612@outlook.com (L.L.); xkr@swfu.edu.cn (K.-R.X.); 2 Key Laboratory for Forest Resources Conservation and Utilization in the Southwest Mountains of China, Ministry of Education, Southwest Forestry University, Kunming 650224, China; 3 Yunnan Plateau Characteristic Agricultural Industry Research Institute, Yunnan Agricultural University, Kunming 650201, China; shuqi_he@foxmail.com

**Keywords:** *Spodoptera frugiperda*, cuticular protein, insecticides, expression pattern, transcriptome

## Abstract

**Simple Summary:**

The fall armyworm (FAW), *Spodoptera frugiperda*, is a notorious agricultural pest worldwide, causing great damage to a wide variety of crops. This pest exhibited a remarkable field-evolved resistance to multiple insecticides. According to the evidence, a few cuticular proteins (CPs) participate in the insecticide resistance of several insects. This study was designed to explore whether CP genes of the FAW exhibit functional roles in responding to insecticides stress. There are a set of CP genes significantly regulated in response to the exposure to different insecticides, implying that CP genes play an important role in the FAW against insecticides stress. The results inspire further functional validation of CP genes in FAWs to gain a better understanding of its resistance to insecticides.

**Abstract:**

The fall armyworm (FAW), *Spodoptera frugiperda*, is a serious pest of crucial crops causing great threats to the food security of the world. It has evolved resistance to various insecticides, while the underlying molecular mechanisms remain largely unknown. Cuticular proteins (CPs), as primary components in cuticle, play an important role in insects’ protection against environmental stresses. Few of them have been documented as participating in insecticide resistance in several insect species. In order to explore whether CP genes of the FAW exhibit a functional role in responding to insecticides stress, a total of 206 CPs, classified into eight families, were identified from the genome of the FAW through a homology-based approach coupled with manual efforts. The temporal expression profiles of all identified CP genes across developmental stages and their responses to 23 different insecticides were analyzed using the RNA-seq data. Expression profiling indicated that most of the CP genes displayed stage-specific expression patterns. It was found that the expression of 51 CP genes significantly changed after 48 h exposure to 17 different insecticides. The expression of eight CP genes responding to four insecticides were confirmed by RT-PCR analysis. The results showed that their overall expression profiles were consistent with RNA-seq analysis. The findings provide a basis for further functional investigation of CPs implied in insecticide stress in FAW.

## 1. Introduction

The fall armyworm (FAW), *Spodoptera frugiperda* (Lepidoptera: Noctuidae), is a notoriously destructive pest that feeds on more than 350 host plants, causing major damage to economically important crops such as corn, rice, cotton, sorghum, soybean and vegetables [1,2]. The FAW is native to tropical and subtropical regions of the Americas. In 2016, it was first reported as an invasive species in Africa [3]. Currently, it has spread to at least 64 nations from Africa, Asia and Australia (https://www.cabi.org/isc/fallarmyworm, accessed on 28 August 2021). Due to its bioecological aspects such as extreme polyphagy, high reproductive capacity, strong migratory behavior and rapid adult dispersion, the FAW poses a global threat to agricultural production and food security. For instance, FAWs have caused maize yield losses of 50% from Africa and southern Asia since 2016 [4], and it was estimated to cause up to $US13 billion per year in crop losses across sub-Saharan Africa [5]. Control of FAWs primarily depends on the use of genetically modified crops expressing *Bacillus thuringiensis* (Bt) toxins and synthetic insecticides [6]. However, the FAW has evolved resistance to Bt crops and a variety of chemical pesticides, including organophosphates, carbamates, pyrethroids, benzoylureas and spinosyns [7,8,9]. The molecular mechanism of resistance is becoming imperative to be investigated to provide insights into the development of resistance management strategies for the FAW.

Cuticular proteins (CPs) are critical constituents of the insect cuticle, the exoskeleton, as well as the cuticle lining the foregut, hindgut and tracheae. They can be classified into more than ten families according to their conserved protein sequence motifs. The most abundant family is known as CPs with the Rebers–Riddiford motif (CPR) that can be assigned into three subfamilies, RR-1, RR-2 and RR-3 [10,11]. The other families include CPAP analogous to peritrophins (CPAP1 and CPAP3), CPs with the Tweedle motif (CPT), CPF with a 44 aa consensus, CPFL (CPF-like) with a conserved C-terminal region similar to CPF but lacking the consensus, CPCFC with C-x (5)-C motifs repeated two or three times, CPs consisting of an 18 aa motif, alanine-rich CPs of low complexity (CPLCA), CPs of low complexity with two invariant glycine residues in the conserved domain (CPLCG), CPs of low complexity with an invariant tryptophan in the conserved domain (CPLCW), proline-rich CPs of low complexity (CPLCP), CPs with well-conserved cysteine residues (CPCFC), glycine rich CP (CPG), CPH comprised of hypothetical CPs and Apidermin restricted to Hymenoptera [11,12,13,14]. As one of the large and diverse families in insects, CPs usually account for about 1% of the protein coding genes in their genomes [15]. Due to the mixture of CPs, they greatly contribute to determine the properties of the insect cuticles for defending against environmental stresses [16,17]. Previous reports have shown that the changed expression of CP genes is associated with the exposure to insecticides, indicating that CPs participate in insecticide resistance [18,19,20,21]. However, the precise roles served by CPs in response to insecticides remain largely unknown.

In this study, CP genes have been annotated based on the recently published chromosome-level genome sequences of FAW [22,23]. They were classified with respect to the families described above and phylogenetically analyzed. We then used high-throughput RNA-seq analysis to reveal diverse CP gene expression patterns across developmental stages and identify the differentially expressed CP genes in response to the treatment of 23 insecticides, followed by validation with a RT-PCR (real time polymerase chain reaction). Combined with genomic and transcriptomic analysis, candidate CP genes of the FAW that are modulated by insecticide exposure were revealed. The data obtained here are helpful for exploring the function of CP genes associated with insecticide stress in this pest.

## 2. Materials and Methods

### 2.1. Gene Identification

To exhaustively identify CP genes from FAWs, the corresponding protein sequences were retrieved from Acyrthosiphon pisum, Anopheles gambiae, Apis mellifera, Bombyx mori, Drosophila melanogaster, Manduca sexta, Pediculus humanus, Spodoptera litura and Tribolium castaneum [12,15,24]. They were used to search against two reported reference genomes of the FAW by Blast with an e-value cutoff of e^−5^ [22,23]. The genomic data of the FAW were downloaded from InsectBase (http://www.insect-genome.com/Sfru/, version WMCG01000000, accessed on 15 May 2021) and CNSA (CNGB Nucleotide Sequence Archive) (https://db.cngb.org/cnsa/, accessed on 23 May 2021) with accession no. CNP0000513. Additionally, putative CP genes were identified by seeking the genomic data based on Hidden Markov Models of different CP families by HMMER3 search (http://hmmer.janelia.org/, accessed on 23 May 2021) [24,25]. The redundant sequences were removed using the CD-HIT program. Then, all identified genes were corrected using the transcriptomic (RNA-seq) data (see below). Predicted CP genes were further classified into families using an online tool, CutProtFam-Pred (http://bioinformatics.biol.uoa.gr/CutProtFam-Pred/, accessed on 20 August 2021) with default parameters [15].

### 2.2. Phylogenetic Tree Construction

Phylogenetic analysis was performed using the corresponding protein sequence of CP genes of FAW, *S. litura*, *B. mori* and *M. sexta*. Amino acid sequences were aligned with MUSCLE in MEGA X [26]. A phylogenetic tree was constructed using the maximum likelihood (ML) method by FastTree 2.1.11 (http://microbesonline.org/fasttree, accessed on 26 July 2021) with SH-like 1000 support under the model of WGA and CAT [27]. Trees were visualized and colored using FigTree v1.4.4 (http://tree.bio.ed.ac.uk/software/figtree/, accessed on 26 July 2021).

### 2.3. Gene Expression Profiling

RNA-seq data of the FAW from different developmental stages including 1st–6th instar larvae, pupae and adults of both sexes were downloaded from InsectBase (http://www.insect-genome.com/Sfru/, accessed on 15 May 2021) [23], which can also be accessed at NCBI with the BioProject accession no. PRJNA590312. To gain the differentially expressed CP genes responding to different insecticides, transcriptomic data from the 3rd instar larvae of FAW exposed to 23 insecticides including four biological, ten single and nine mixed chemical insecticides were downloaded from CNSA (https://db.cngb.org/cnsa/, accessed on 23 May 2021) with accession no. CNP0001020 [22]. The detailed information of the pesticides can be obtained from Gui et al. [22]. These pesticides were commonly used in agricultural production. Based on these data, a pipeline implemented in TBtools was used to calculate the transcript abundance with the TPM/FPKM method [28]. Significantly differentially expressed CP genes were tested using DESeq2 [29]. Gene expression profiles were illustrated and hierarchically clustered using the HeatMap package in TBtools [28].

### 2.4. RT-PCR Analysis

FAW used in the experiments were a colony maintained in our lab. Its larvae were fed on corns. The 3rd larvae of the FAW exposed to four insecticides including 20% dinotefuran soluble granules (P10), 33 g/L avermectin and bifenthrin emulsifiable concentrate (P14), 40% bifenthrin and thiacloprid suspension concentrate (P16) and 35% chlorantraniliprole water dispersible granule (P23) were treated as described by Gui et al. [22]. In brief, square pieces of corn leaves (2 × 2 cm) were dipped into different insecticides according to the Insecticide Resistance Action Committee (2019) (https://irac-online.org/, accessed on 5 March 2019). They were put on moist filer paper in a Petri dish with a diameter of 10 cm for feeding the larvae of the FAW. Ten FAW larvae were added to each Petri dish. The larvae fed on the corn leaves dipped into solution was set as control. Corn leaf pieces were replaced at an interval of 12 h. After 24 and 48 h, ten surviving larvae were put together for one biological replicate. They were transferred into a 1.5 mL Eppendorf tube. After homogenization of samples in liquid nitrogen, their total RNA was extracted with TRIzol regent (Invitrogen, Carlsbad, CA, USA) according to the manufacturer’s protocol. Its quantity and integrity were accessed by Nano Drop ND-1000 spectrophotometer (PeqLab, Erlangen, Germany) and 1% formaldehyde agarose gel electrophoresis, respectively. An equal amount of 1 ug total RNA from each sample was used to synthesize cDNA using PrimeScript RT Reagent Kit with gDNA Eraser (TaKaRa, Dalian, China) following the manufacturer’s protocol. A total of eight CP genes were selected for validating their expressions after exposure to the above four insecticides by RT-PCR with Dream Taq Green PCR Master mix (Thermo Fisher Scientific, Waltham, MA, USA). Gene-specific primers were designed based on their sequences derived from the genome of the FAW using Primer Premier 5. Actin genes were used as an internal reference gene for normalization. Primers used here were listed in Table S1. PCR conditions were as follow: 3 min at 95 °C, 36 cycles consisting of 95 °C for 30 s, 54 °C for 30 s and 72 °C for 1 min, followed by 72 °C for 10 min. PCR products were viewed in 1% agarose gel stained with Gel Red (Biosharp, Hefei, China).

## 3. Results

### 3.1. Identification and Characterization of CP Genes

**Figure 1:**
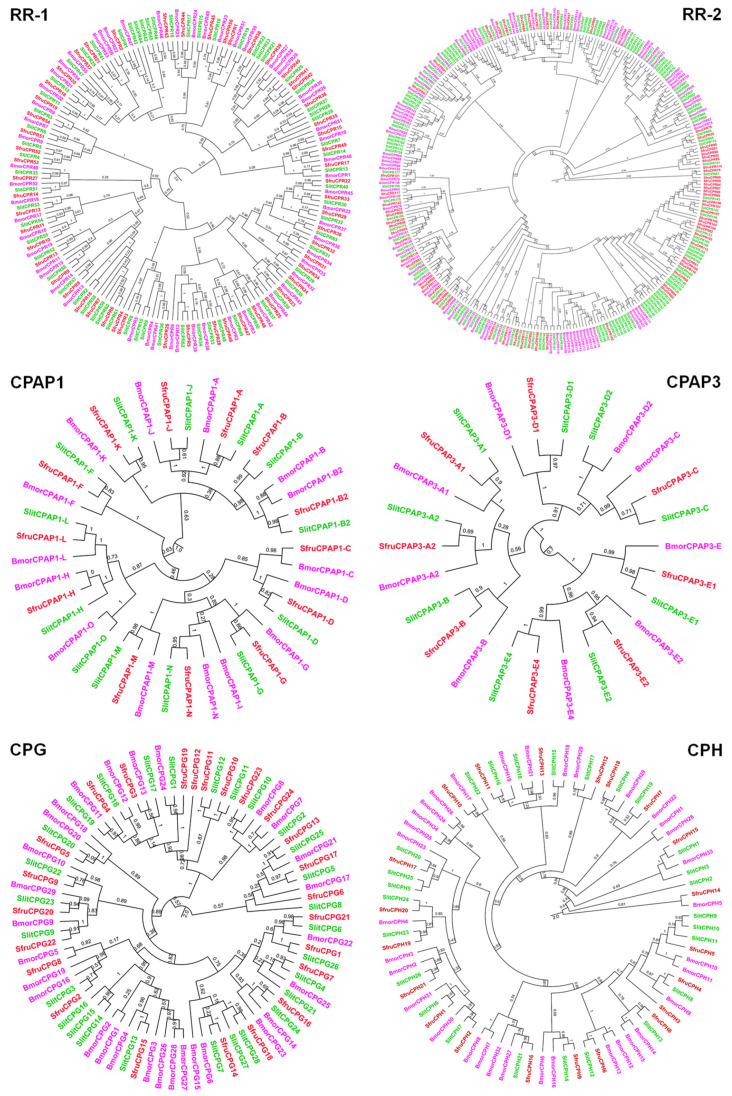
Maximum-likelihood tree of candidate CPs belonging to different families from four lepidopteran species. The tree was constructed by FastTree v2.1.11 (http://microbesonline.org/fasttree, accessed on 26 July 2021), based on an aligned amino acid sequence with MUSCLE in MEGA X. CPs marked by the same colors represents those from the same species. Bmor, *Bombyx mori*; Msex, *Manduca sexta*; Sfru, *Spodoptera frugiperda*; Slit, *Spodoptera litura*.

A total of putative 206 CP genes were identified by searching against the FAW genome followed by manual confirmation and correction (Table 1 and Table S2). They were classified into eight families including RR, CPT, CPFL, CPLCA, CPCFC, CPAP, CPG and CPH, based on CutProtFam-Pred and phylogenetic analyses. Among them, CPR comprised 124 members and constitutes the largest CP family in the FAW, followed by CPG and CPH with 24 and 21 members, respectively. The CPR gene family consisted of three subfamilies including RR1, RR2 and RR3. For the RR-1 and RR-2 subfamilies, they had the largest number of genes with 54 and 69, respectively. On the basis of sequence similarity to the known RR-3 proteins, only one CP gene assigned into the RR-3 subfamily was identified in the FAW genome. Within the CPAP family, 13 and 8 genes were classified into CPAP1 and CPAP3 subfamilies, respectively. To compare the FAW CPs with *S. litura*, *B. mori* and *M. sexta* CPs, phylogenetic tree of major CP families or subfamilies was constructed separately (Figure 1). The results showed that RR-1 and RR-2 proteins were clearly separated. CPs from these four lepidopteran species were largely orthologous to each other, displaying an evolutional conservation. In most of the clades, each species had a similar number of genes. A FAW-specific clade was rare in the phylogenetic trees. Only several FAW-specific clades were observed in the RR-2 subfamily, indicating species specific gene expansions by duplication.

### 3.2. CP Gene Expression across Developmental Stages

**Figure 2:**
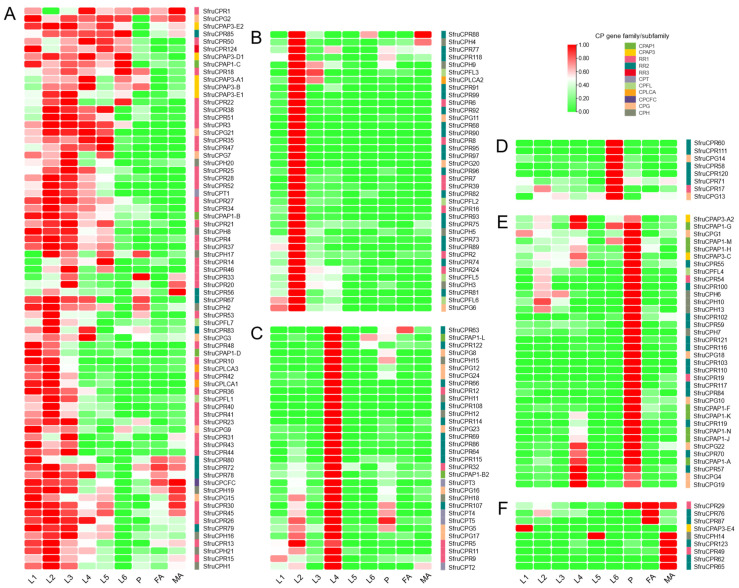
The expression profiles of CP genes across developmental stages. (**A**–**F**) highlight the hierarchically clustered CP genes with stage-specific expression patterns. The heat map was illustrated using the TPM values of CP transcripts calculated with the RNA-Seq data. Red and green indicate the high and low expressions, respectively. L1–L6: 1st–6th instar larvae; P, pupae; FA, female adult; MA, male adult.

The expression profiles of CP genes during FAW development were determined by using transcriptomic data from different developmental stages including 1st–6th instar larvae, pupa and adult (Table S3). There were ten CP genes with no TPM values observed for all samples, indicating that their expressions were not detected in this way. The expressions of other CP genes, represented by TPM values, were subjected to hierarchical clustering analysis. The results revealed that CP genes dynamically expressed across different developmental stages, represented by six distinct groups (Figure 2). More than half of the CP genes clustered into group A showed high expressions in 1st–3rd instar larvae. In this group, part of the CP genes also highly expressed in other stages. CP genes in groups B, C and D observed high expressions in the 2nd, 4th and 6th instar larvae, respectively. CP genes in group E highly expressed in pupae, part of which also showed high expression in the 4th instar larvae. In group F, CP gene expressions were abundant in adults, which were related to sex-biased expressions except for the SfruCPR29 gene. Additionally, CP genes that show sex-biased expressions can be found in other groups. Interestingly, most RR-1 genes expressed extremely highly at the larval stage, especially in the 1st–2nd instar larvae (Figure 2 and Figure S1). Besides the 1st instar larvae, most RR-2 genes displayed stage-specific abundant expression at other stages. Additionally, stage-specific expression patterns were observed for some CP genes from other families. It should be noted that some CP genes from different families had high levels of expression at nearly all life stages, e.g., SfruCPR1, SfruCPAP3-E2 and SfruCPG2, which represents a small number of all CP genes.

### 3.3. Influence of Insecticides on CP Gene Expression

**Figure 3:**
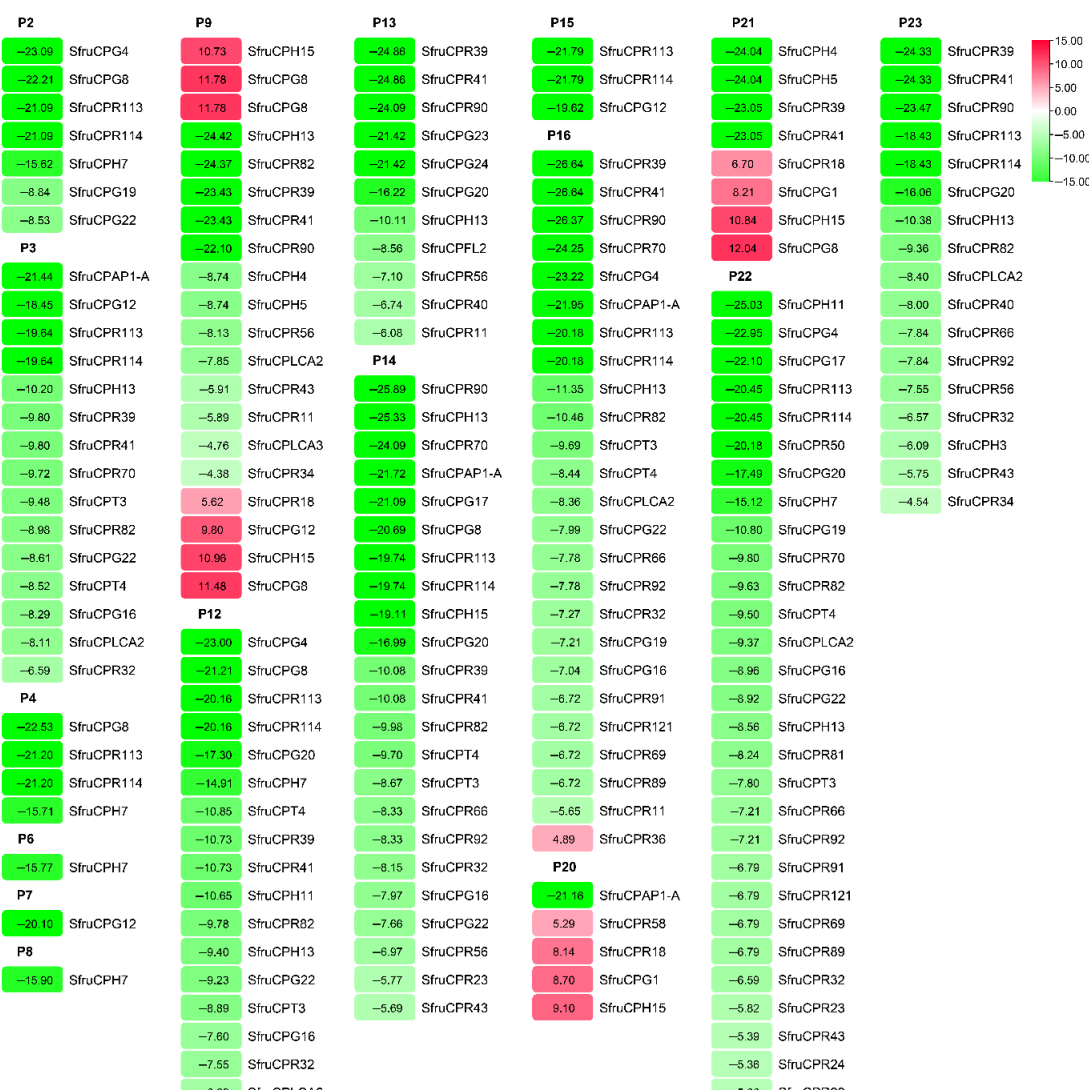
The detailed fold changes of CP genes after exposure to different insecticides. The detailed information of different insecticides corresponding to their codes (above the heat map) are represented in Table 2. Transcriptional levels were expressed as mean fold changes to the control. The red and green colors represent the up and down regulation of CP gene expression by different insecticides, respectively. The detailed fold change values on the left and the differentially expressed CP genes on the right are shown, which are below the insecticide codes.

**Figure 4:**
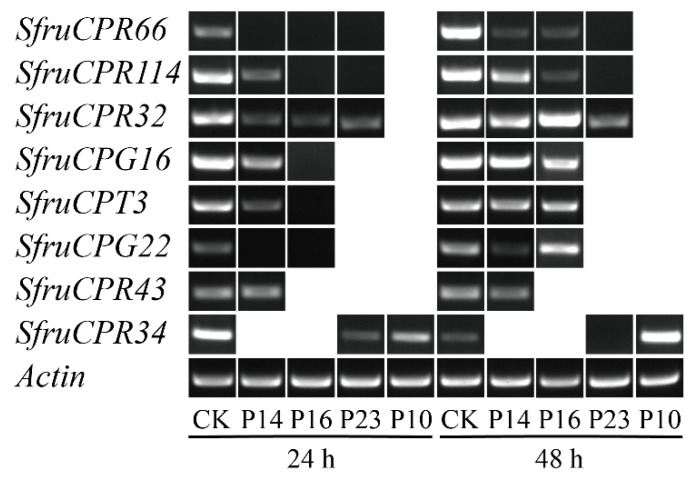
Relative expression level of eight candidate CP genes after exposure to four different insecticides. The expression of CPs was analyzed by RT-PCR. The actin gene was used as an internal reference. The detailed information of four different insecticides corresponding to their codes (P10, P14, P16 and P23) are represented in Table 2. The 3rd instar larvae of FAW exposed to different insecticides after 24 h and 48 h were used for RT-PCR analysis. CK represents no treatment of insecticides. It should be noted that if the expression of selected eight candidate CP genes do not change after exposure to these insecticides through RNA-seg analysis, their expressions are not further validated by RT-PCR analysis. Thus, the missing data for some treatments at each timepoint (24 h or 48 h after treatment) indicate that the CP gene expression was not validated by RT-PCR analysis.

By comparative transcriptome analysis, it was found that the expression of 51 CP genes in the 3rd instar larvae of FAWs significantly changed after exposure to 23 insecticides in comparison to the control (Table 2 and Table 3). The exposure of six insecticides did not influence the CP gene expression. The exposure of P22 led to the largest number of CP genes with changed expression, followed by P16 and P14. Among the differentially expressed CP genes, most of them displayed 4.38–26.64-fold down regulation (Figure 3). Seven (SfruCPR18, SfruCPR36, SfruCPR58, SfruCPG1, SfruCPG8, SfruCPG12 and SfruCPH15 genes) were defined as up regulated with the fold change ranging from 4.89 to 12.04, which were induced by five insecticides. In order to validate the transcriptomic data, eight CP genes differentially expressed after exposure to four insecticides including P10, P14, P16 and P23, were selected for further verification using RT-PCR (Figure 4 and Figure S2). Regarding those genes down regulated by P10, P14, P16 and P23 in RNA-Seq data, all CP genes were down regulated at 24 h post exposure to these insecticides. Besides SfruCPR32, SfruCPG16 and SfruCPT3 genes in the samples treated by P14 and P16, SfruCPG22 in samples treated by P16, and SfruCPG34 in samples treated by P10, the other CP genes were remarkably down regulated at 48 h after exposure to the above four insecticides, of which their overall expression profiles of CP genes were consistent with RNA-seq analysis.

CP genes differentially expressed after exposure to different insecticides are listed in the left column. Different insecticides corresponding to their codes in Table 2 that lead to the change of CP gene expression are listed in the right column.

## 4. Discussion

The present report provides the first genome-wide identification of the CP gene families in the FAW, a voracious agricultural pest causing considerable economic costs [5]. The number of CP genes in FAWs was consistent with previous reports that they are generally comprised of around 1% of the genes encoding in the genome of insect species [15]. Compared with the number of CP genes in genomes of other lepidopteran species, CP genes carried by FAWs are lower than those in the genomes of *S. litura*, *B. mori* and *M. sexta* [12,15,24,30]. The CP gene number is significantly varied across insect species and taxa, ranging from 45 in *A. mellifera* to 305 in *Aedes aegypti* [31,32,33]. Interestingly, genome-based analysis revealed that 287 putative CPs were predicted in *S. litura*, a lepidopteran species closely related to the FAW [12]. The high number of annotated CPs in *S. litura* results in a large species-specific expansion of RR-1 and RR-2 CPs by gene duplication events. The number of CP genes in FAWs is obviously distinct from *S. litura*, suggesting a high rate of gene turnover between these two species. Similar to other reports, phylogenetic analysis of major CP families revealed that co-orthologous groups were present in FAW, *S. litura*, *B. mori* and *M. sexta* [12,24]. The orthologous CPs from different species clustered in a monophyletic clade are attributed to gene duplication events occurring after speciation [24,34]. It suggests that CP gene families evolved from an ancestral gene through duplication and diversification.

Hierarchical clustering analysis indicated that six clusters of CP genes with dynamic expression profiles were strikingly observed across developmental stages. Stage-specific expression of CP genes was also detected in other insects, such as *Microplitis mediator*, *Bactrocera dorsalis*, *Dendrolimus punctatus* and *Nilaparvata lugens* [14,31,34,35]. During larval to pupal development of FAWs, the expression pattern of the majority of CP genes had major changes. In addition, a much larger number of CP genes showed high expression in larvae and pupae than in adults. These should be due to the production of new cuticles associated with metamorphosis requiring large expression of CP genes. Similar to *D. punctatus*, the majority of CP genes from different families were observed in high abundance at a specific life stage [34]. However, it is difficult to speculate whether the stage-specifically expressed CP genes entirely determine the cuticle formation during metamorphosis. Few CP genes were abundant in nearly all life stages, suggesting a significant role in general cuticle synthesis. It was documented that CP genes that displayed the most abundant expression in specific tissue at particular developmental stages played important roles associated with such tissues during specific developmental periods [12,14,24,31,33,34]. Thus, further investigation into unravelling the expression of CP genes between pre-molt/eclosion and post-molt/eclosion at particular developmental stages and multiple tissues is essential to provide deep insights into the functional perspectives of CPs in FAW.

As crucial components in insect cuticular, CPs functioned in environmental stresses such as insecticides, extreme temperature and ultraviolet by mediating cuticular permeability [17]. By comparative RNA-Seq analysis, it was revealed that the expression of CP genes was in response to exposure to six insecticides, suggesting a role in the adaption to some insecticides in FAWs. Similarly, CP genes responding to insecticides have been identified in a few insects. For example, the transcripts of two CPG genes (*Ld-GRP1* and *Ld-GRP2*) were highly induced by azinphosmethyl in adult of *Leptinotarsa decemlineata* [36]. Koganemaru et al. [37] found that many CPR genes were upregulated in pyrethroid resistant bed bug (*Cimex lectularius*). The expression of CPAP3-A1, CPAP3-C1, CPAP3-D1 and CPAP3-E2 genes were significantly induced in the oriental fruit fly (*Bactrocera dorsalis*) by malathion, the main pesticide used against this pest [31]. Interestingly, it has been documented that CP genes were over-expressed in deltamethrin-resistant strains of *A. sinensis* and *Culex pipiens pallens*, and pyrethroid-resistant strains of *A. stephensi*, *A. gambiae* and *A. sinensis* [38,39,40]. This indicated that the upregulation of CP genes is linked to insecticide resistance, while only a few of them were evidenced as involved in such a role. For instance, the expression of the CpCPLCG5 gene in *C. pipiens pallens* was higher in the deltamethrin-resistant strain than in the susceptible strain [19], and knockdown of the CpCPLCG5 gene increased the susceptibility of the deltamethrin-resistant strain [41]. The over-expression of CP genes in resistant strains led to the increased thickness of cuticle, resulting in a cuticular penetration resistance to insecticide. Due to CP genes that exhibit higher expressions in resistant strains than in susceptible strains with the most potential to be implicated in insecticide resistance [19,21,36,42], CP genes of FAW up regulated here are worth being focused on to investigate their role in insecticide resistance. In addition to induction, CP genes can also be reduced by insecticides. It is similar to detoxifying enzyme genes such as cytochrome P450, glutathione S-transferase and carboxylesterase genes being down regulated by insecticides, while the exact roles of the suppressed detoxification enzyme genes in insecticide resistance remain largely unknown [43]. For instance, among the 68 CP genes differentially expressed after 6, 24 and 48 h exposure to permethrin in *A. stephensi*, part of them were down regulated [44]. Since silencing or mutation of CP genes can influence the development, and lead to the reduction of resistance to environmental stresses including insecticides [17,35], the reduced expression of CP genes would enhance the penetrability of insecticides by decreasing the thickness of cuticle. The CP genes associated with down-regulated expressions in FAWs could deserve to be potential candidates for further investigation of their important role in response to insecticide stress.

## 5. Conclusions

Genome-wide analysis with exhaustive homology-based searches and manual efforts in FAWs led to the identification of 206 CP genes. Their expression profiles during different development time points and in response to the exposure of 23 different insecticides were investigated. A set of them, presumed to be responsive to a range of insecticides, were identified and validated using RT-PCR analysis. Our results provide insights for the further functional investigation of CP genes of FAW involved in insecticide stress.

## Figures and Tables

**Table 1:** Summary of the gene number of each cuticular protein family in *Spodoptera frugiperda*, *S. litura*, *Bombyx mori* and *Manduca sexta*.

Motif	*S. frugiperda*	*S. litura* ^a^	*B. mori* ^b^	*M. sexta* ^c^
RR-1	54	63	56	79
RR-2	69	129	93	124
RR-3	1	1	3	4
CPT	5	5	4	4
CPF	0	1	1	1
CPFL	7	7	4	6
CPLCA	3	4	2	0
CPCFC	1	1	1	1
CPAP1	13	13	14	15
CPAP3	8	9	9	10
CPG	24	28	29	0
CPH	21	26	31	0
Total	206	287	247	248

Sources of the numbers: ^a^ Liu et al. [12]; ^b^ Futahashi et al. [30]; ^c^ Dittmer et al. [24]. The gene number of *M. sexta* does not include the 18 aa family.

**Table 2:** Differentially expressed CP genes of *Spodoptera frugiperda* after exposure to 23 insecticides.

Insecticide Code	Insecticide Treatment	Differentially Expressed CP Gene		
		Up	Down	Total
P1	80 billion spores/mL *Metarhizium anisopliae* suspension concentrate	0	0	0
P2	100 billion spores/g *Bacillus thuringiensis suspension* concentrate	0	7	7
P3	100 billion spores/mL *Empedobacter brevis* suspension concentrate	0	15	15
P4	0.3% azadirachtin emulsifiable concentrate	0	4	4
P5	3% emamectin benzoate microemulsion	0	0	0
P6	15% indoxacarb suspension concentrate	0	1	1
P7	19% spinetoram water dispersible granule	0	1	1
P8	10% chlorfenapyr suspension concentrate	0	1	1
P9	10% ethofenprox suspension concentrate	2	0	2
P10	20% dinotefuran soluble granules	4	13	17
P11	25% cyhalodiamide and clothianidin suspension concentrate	0	0	0
P12	8% avermectin and indoxacarb water dispersible granule	0	17	17
P13	30% hexaflumuron and indoxacarb suspension concentrate	0	11	11
P14	33 g/L avermectin and bifenthrin emulsifiable concentrate	0	23	23
P15	34% spinetoram and methoxyfenozide suspension concentrate	0	3	3
P16	40% bifenthrin and thiacloprid suspension concentrate	1	25	26
P17	10% emamectin and indoxacarb suspension concentrate	0	0	0
P18	12% emamectin and flutolanil microemulsion	0	0	0
P19	40% chlorantraniliprole and thiamethoxam water dispersible granule	0	0	0
P20	50 g/L lufenuron emulsifiable concentrate	4	1	5
P21	10% cyantraniliprole suspension concentrate	4	4	8
P22	240 g/L metaflumizone suspension concentrate	0	29	29
P23	35% chlorantraniliprole water dispersible granule	0	17	17

**Table 3:** Detailed list of differentially expressed CP genes after exposure to different insecticides.

Differentially Expressed CP Gene	Insecticide Code
SfruCPR50, SfruCPR81, SfruCPR24, SfruCPR29	P22
SfruCPR121, SfruCPR69, SfruCPR89, SfruCPR91	P16, P22
SfruCPFL2, SfruCPG24, SfruCPG23	P13
SfruCPG16, SfruCPT3, SfruCPT4	P3, P12, P14, P16, P22
SfruCPH5, SfruCPH4	P10, P21
SfruCPG17, SfruCPR23	P14, P22
SfruCPR92, SfruCPR66	P14, P16, P22, P23
SfruCPR41, SfruCPR39	P3, P10, P12, P13, P14, P16, P21, P23
SfruCPR114, SfruCPR113	P2, P3, P4, P12, P14, P15, P16, P22, P23
SfruCPLCA3	P10
SfruCPR36	P16
SfruCPR58	P20
SfruCPH3	P23
SfruCPR34	P10, P23
SfruCPH11	P12, P22
SfruCPR40	P13, P23
SfruCPG1	P20, P21
SfruCPG19	P2, P16, P22
SfruCPR11	P10, P13, P16
SfruCPR18	P10, P20, P21
SfruCPG4	P1, P12, P16, P22
SfruCPR56	P10, P13, P14, P23
SfruCPR90	P10, P13, P14, P16, P23
SfruCPR43	P10, P14, P22, P23
SfruCPG12	P3, P7, P10, P15
SfruCPAP1-A	P3, P14, P16, P20
SfruCPR70	P3, P14, P16, P22
SfruCPH15	P9, P10, P14, P20, P21
SfruCPG20	P12, P13, P14, P22, P23
SfruCPG22	P2, P3, P12, P14, P16, P22
SfruCPH7	P2, P4, P6, P8, P12, P22
SfruCPLCA2	P3, P10, P12, P16, P22, P23
SfruCPR32	P3, P12, P14, P16, P22, P23
SfruCPG8	P2, P4, P9, P10, P12, P14, P21
SfruCPR82	P3, P10, P12, P14, P16, P22, P23
SfruCPH13	P3, P10, P12, P13, P14, P16, P22, P23

## Data Availability

The data presented in this study are available in the Supplementary Materials section.

## Supplementary Materials

The following are available online at https://www.mdpi.com/article/10.3390/insects12110997/s1: Table S1: RT-PCR primers used in this study; Table S2: List of CP genes identified in the FAW genome; Table S3: TMP values of CP genes of FAWs at different developmental stages obtained from the RNA-seq data; Figure S1: The expression profiles of CP genes from different families or subfamilies at different developmental stages. The heat map was illustrated using the TPM values of CP transcripts calculated with the RNA-Seq data. Red indicates the high expression and green indicates the low expression; Figure S2: Validation of expression profiles of CP genes after exposure to four different insecticides by RT-PCR. The expression data represent the quantification of the band in Figure 4 from each sample, which have been normalized to the reference gene, actin. The data was statistically analyzed using the SPSS program.
