# Supplementary material for: Genomic and Transcriptomic Analysis Reveals Cuticular Protein Genes Responding to Different Insecticides in Fall Armyworm Spodoptera frugiperda
Source: Insects. 2021 Nov 5;12(11):997. doi: 10.3390/insects12110997 (PMC8622913; doi:10.3390/insects12110997)
Supplement: Supplementary file 1 [file insects-12-00997-s001.zip › Table S1.pdf]

Table S1. RT-PCR primers used in this study.

Gene	Sense primer (5'-3')	Anti-sense primer (5'-3')
SfruCPR32	GCCTGCTGAGCAGAACTACAAC	GACCTTTCTGGATTTCGGGAG
SfruCPR34	TCGTAGTTCTGTCCCTGCTCG	CAGCGATGTAGGAGACGGAGAT
SfruCPR43	GTATTCGCTCTCTTTGCCGG	GAGCAGGGACAACTCGAAGG
SfruCPR66	CAATGGATTCAACGCGGTAG	GCTTGATCTTCTGCTGGGGT
SfruCPR114	AGCCAACGGACAAGAAGGAC	TGGGCTCGTCGTGGAAAT
SfruCPT3	ATAGTACTCGCCTGCGTGGC	GCTGCTCTTCGGGTTTCTTG
SfruCPG16	CACTCCGAAAGCGAGGGTT	CCGATGGTGGTGTAGTGAGAAG
SfruCPG22	GTGAAGAAAGAGCACAGCCATC	TTGTAGCCCTCGCCAGAGTC
Actin	GGCATTGTAGTGGACAGTGGTG	AGCATTGGAGAACTCAGGGTG
