# Supplementary figures and images for: Genomic and Transcriptomic Analysis Reveals Cuticular Protein Genes Responding to Different Insecticides in Fall Armyworm Spodoptera frugiperda
Source: Insects. 2021 Nov 5;12(11):997. doi: 10.3390/insects12110997 (PMC8622913; doi:10.3390/insects12110997)

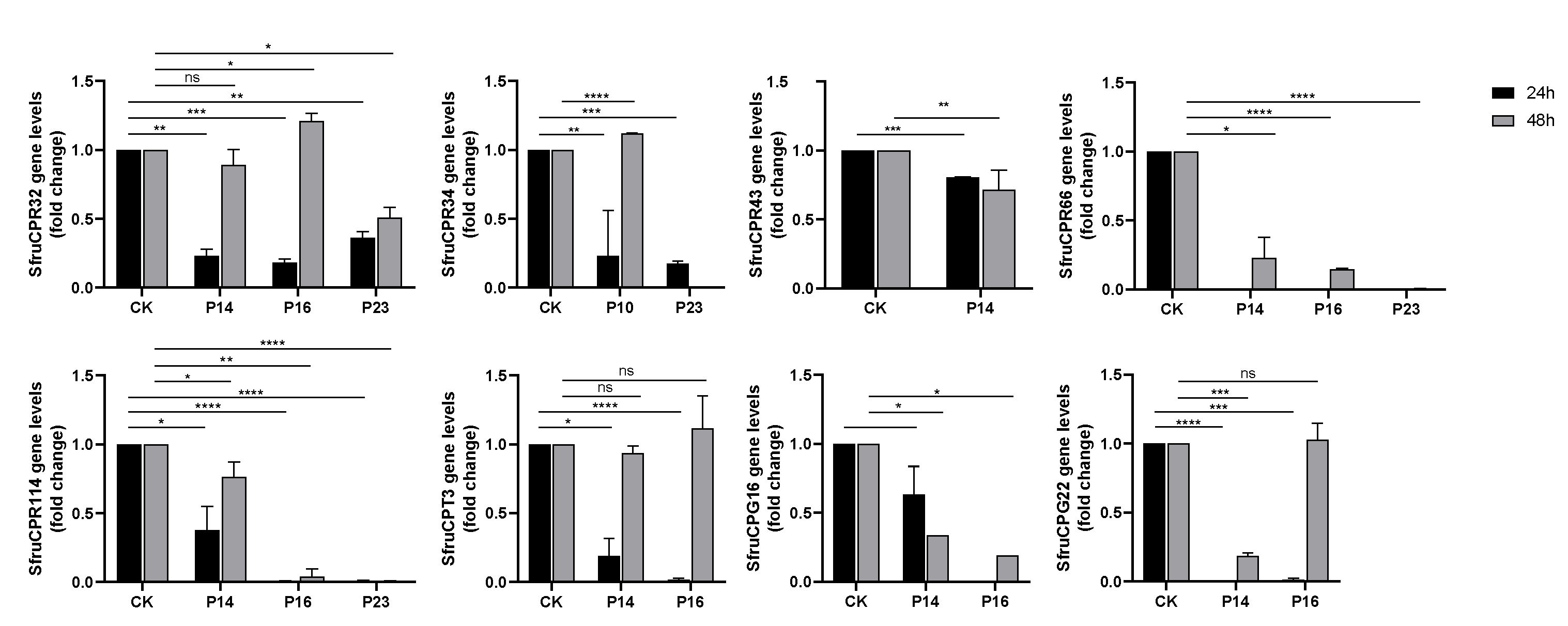

Supplement: Supplementary file 1 [file insects-12-00997-s001.zip › Figure S2.tiff]
